# Early laparoscopic Kasai's procedure in a low weight newborn

**DOI:** 10.4103/0972-9941.33276

**Published:** 2007

**Authors:** M Lopez, N Kalfa, D Forgues, M P Guibal, R B Galifer, H Allal

**Affiliations:** Department of Visceral Pediatric Surgery, Lapeyronie Hospital, Montpellier, France

**Keywords:** Biliary atresia, Kasai's procedure, laparoscopy, newborn

## Abstract

The authors present an early laparoscopic treatment in a newborn with biliary atresia. They describe the technical details of the Kasai laparoscopic procedure. A 10-day-old girl, weight 2.4 kg, was admitted with a history of jaundice and fecal acholia since birth, with elevated total bilirubin and abnormal hepatic test. Abdominal ultrasound showed a small gallbladder with hyperechogenicity in porta hepatis and absence of biliary principal duct. Other metabolic and hematological tests were normal. The procedure was performed at 20-day-old by laparoscopy. The cholangiography confirmed the biliary atresia and Kasai's procedure was continued by laparoscopy and transumbilical extracorporeal Roux-Y approach. The duration of the procedure was 220 min, with good tolerance of pneumoperitoneum due to the laparoscopy. Feedings of breast milk began on the third day postoperative, presenting normal colored stools, with normalization of the hepatic test. A 20 months follow-up was without complications.

## INTRODUCTION

Before the introduction of the Kasai porto-enterostomy, biliary atresia survival rates were less than 5% at 12 months of age.[1] Survival rates greater than 50% after porto-enterostomy have been reported and with the addition of liver transplantation, survival rates greater than 90% should be now expected.[2]

The major determinant factors of a satisfactory outcome following porto-enterostomy are: the patient's age at operation (under three months), the successful establishment of postoperative bile flow, the presence of microscopically demonstrable ductal structures in the hilum, the degree of parenchyma disease at diagnosis and technical factors of the anastomosis and diminish of jaundice and hepatic blood-test. Standard operation includes large, painful, muscle-cutting laparotomy to accomplish hilar dissection and porto-enterostomy according to Kasai technique or variants.[3] In addition children with biliary atresia are frequently operated upon some degree of malnutrition and hepatic dysfunction and they show a high rate of peroperative complications due to surgical trauma.[4]

Laparoscopic instrumentation and new techniques have advanced. After first report by Esteves, the umbilical approach could be used for safe extracorporeal Roux-en-Y enteric anastomosis in babies with laparoscopic assistance.[5] We report that Kasai porto-enterostomy can be performed in a low weight newborn by laparoscopy without complications. The initial result was encouraging and the patient recovered promptly after this procedure.[6]

## CASE REPORT

A 10-day-old girl, weight 2.5 kg, was admitted with a history of jaundice, choluria and fecal acholia since birth. The pregnancy had been uneventful and the mother had addiction to morphine. All serologic studies were normal; she was born by normal delivery at 39 weeks' gestation weighing 2.3 kg. Laboratory data by the age of 12 days showed that total bilirubin (Tbil) was 26.4 mg/dl (normal 0.2-1.0); direct bilirubin (Dbil), 12.5 mg/dl; alanine aminotransferase (ALT) 94 IU/l (normal 5-32); aspartate aminotransferase (AST) 90IU/l (normal 9-32); alkaline phosphatase (AP) 153 IU/l; and gamma-glutamyltransferase (GGT) 180 IU/l (normal 5-35). Other metabolic, hematological and neonatal serologic test results were normal. An ultrasound scan (US) showed a small gallbladder, no intra or extra hepatic bile ducts, with hyperechogenicity at level hepatis portal that suggested the possibility of biliary atresia. At 20 days of age, a laparoscopic exploration was elected.

The procedure was performed with the patient positioned at the foot of the table, the surgeon at the patient's feet; the assistant with the camera at the right, the second assistant and instrument nurse at the left.

Anesthesia was performed with inhaled and intravenous agents associated with short acting intravenous opiates and muscle relaxants were administered to facilitate the surgical procedure. The patient was intubated and mechanically ventilated with the inspired oxygen fraction setting at 70%.

A 7 mm incision was made in the upper umbilical ring by open technique for the telescope trocar position. The CO^2^ was insufflated to 8 mmHg. Four additional 3.0 mm trocars were placed under direct laparoscopic vision in the left and right upper quadrant, left iliac fossa and epigastric area.

**Figure 1:**
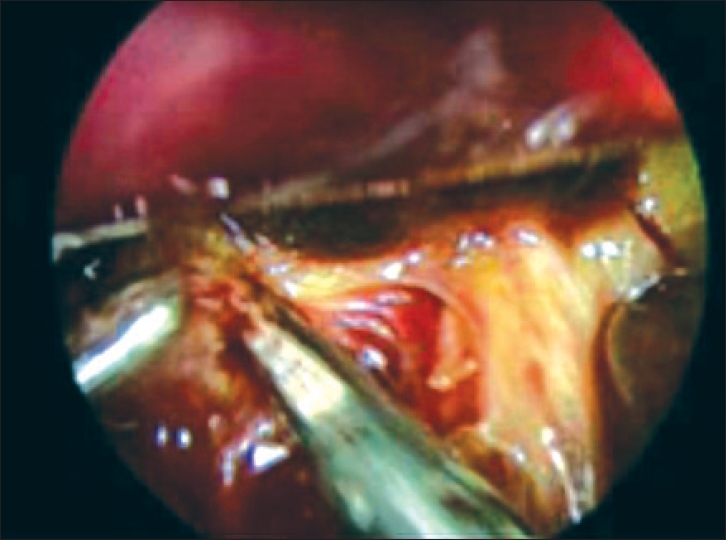
Porta hepatis dissection

The examination confirmed a liver cirrhotic appearance an extra hepatic bile-duct atresia by cholangiography with a small, fibrotic gallbladder without passage into biliary duct. For liver exposure we used the palpator in epigastric area which rotates upward to expose the porta hepatis (Figure 1). Thereafter, mobilization of the gallbladder following the cystic duct, to the junction with the extra-hepatic duct remnants was performed. The reliquant was divided distally and then dissected toward the hepatic hilum, just to find the division of the portal vein and the hepatic arteries' entry point. This exposition allowed a complete transection of “porta hepatis” just under the liver surface with scissors leaving a surface with ducts draining bile [Figure 1].

With the epigastria trocar the left colon was up forward and the jejunum was secured 20 cm from Treitz ligament and drawn to the umbilical port under direct vision and withdrawn through the umbilicus. The pneumoperitoneum was interrupted to relieve tension on the mesentery as necessary to make extracorporeal transumbilical jejunal Roux-en-Y anastomosis. The jejunum was transected by stapler. Another 40 cm segment beyond the distal was selected for lateral-end anastomosis and was placed back into the abdomen. The correct position of the intestine was checked by laparoscopy.

**Figure 2:**
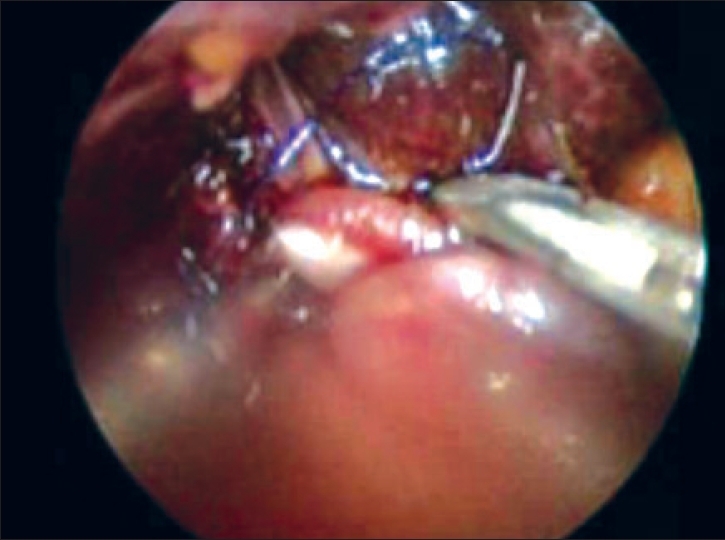
Biliary-digestive anastomosis

The tied proximal jejunum was passed through the mesocolon and the lateral-end running suture around the porta hepatis was done using intracorporeal self-block sutures posterior and running suture anteriorly [Figure 2]. The percutaneus liver biopsy by hepafix needle was associated.

The tolerance of pneumoperitoneum was acceptable. The operative time was 220 min. The infant needed no respiratory support postoperatively. Feeding began on the third day, producing normal colored stools on the same day.

Broad-spectrum antibiotics were continued for only three days postoperative and when oral feedings began, amoxicillin and clavulonic was given orally. The jaundice progressively declined with normalization hepatic function with a follow-up of 20 months.

## DISCUSSION

Prolonged neonatal jaundice may be caused by different medical and surgical pathologies. Extra hepatic biliary atresia (EHBA) is one of the causes.[7] Early diagnosis of EHBA in jaundiced infants is mandatory, because the success rate of the operation is inversely proportional to the age of the patient, 80% if the operation is performed when the infant is younger than 60 days, but the success rate decreases 20% if the child is older than three months of age.[8,9] The diagnostic investigations have to be accelerated and the admittance of the patient to the surgical unit must not be delayed. The evaluation of an infant with prolonged jaundice consists of biochemical and serologic tests and abdominal ultrasonography. The positive “triangular cord sign” in the abdominal ultrasonography is highly suggestive of EHBA,[10] hepatobiliary scintigraphy and fine-needle liver biopsy is carried out.[6,11,12]

Laparoscopic instrumentation and techniques have advanced to the point that laparoscopic, has found a great spectrum of indications in pediatric surgery and now it is used also in biliary atresia cases.[13] Before the introduction of laparoscopy, laparotomy had to be performed on the infants with prolonged jaundice in whom neonatal hepatitis could not be confirmed with the laboratory, radiology and histopathology investigations. Conventional surgical procedures in biliary atresia, used to gain some bile drainage into the intestine in children; have been achieved through large laparotomy in the upper abdomen. One of the largest incisions in pediatric abdominal surgery, with possible complications related to this approach include: pains, breathing limitation leading to pulmonary complications, nerves damage, prolonged ileus, wound dehiscence, large or recurrent incision hernias, rib damage, peritoneal adhesions. Dehiscence and hernias are usually presented in patients with hypoproteinemia, ascitis, which are common features in these patients.[7] When the procedure was started laparoscopically, its utility was limited only to evaluate the liver status: for irregular liver surface and greenish-brown color in patients with EHBA. The presence or absence of the gallbladder, as well as biliary ducts is obviously atretic by cholangiography. Progressive improvements in instrumentation over the last 15 years have led to the development of 3 mm, short laparoscopic instruments designed specifically for use in the pediatric population allowing new techniques and new complex procedures.

The original technique reported by Esteves showed a new approach for Laparoscopic hilar dissection, extracorporeal entero-anastomosis and porto-enterostomy with good results. Today this procedure could decrease theses complications with advantages of minimally invasive technique, safe, short-lasted, the intraabdominal viscera are minimally touched, the opening is small,[14–15] showing significantly lower incidences of adhesions.[16] Amplified, well-illuminated visibility of the tiny hilar structures is guaranteed by laparoscopy. We believe that laparoscopic porto-enterostomy and extracorporeal transumbilical jejunal anastomosis is a promising technique that may be performed in a low weight newborn without complications, allowing the patient to recover promptly. The initial results have been encouraging and its real impact will be revealed after additional cases. Theses procedures should be performed by a surgeon experienced both in biliary surgery and advanced laparoscopic to increase the chances for successful results.[17]
